# Comparative analysis of Chinese classical prescriptions and global traditional polyherbal formulations: insights from the database of global polyherbal formulation (GPFD)

**DOI:** 10.1186/s13020-025-01145-7

**Published:** 2025-10-07

**Authors:** Hongguo Chen, Zhaoyu Liu, Yupeng Du, Xiangxiao Meng, Liangliang Xue, Xiang Sun, Zhuangyuan Xie, Liping Chen, Fan Li, Ruolan Du, Jingwei Zhou, Ting Wang, Liang Leng, Shengpeng Wang

**Affiliations:** 1 State Key Laboratory of Quality Research in Chinese Medicine, Institute of Chinese Medical Sciences, University of Macau, Macao, 999078 China; 2 Institute of Herbgenomics, Chengdu University of Traditional Chinese Medicine, Chengdu, 611137 China; 3 Key Laboratory of Saline-alkali Vegetation Ecology Restoration (Northeast Forestry University), Ministry of Education, Harbin, 150040 China; 4 State Key Laboratory for Quality Ensurance and Sustainable Use of Dao-di Herbs, Key Laboratory of Beijing for Identification and Safety Evaluation of Chinese Medicine, Institute of Chinese Materia Medica, China Academy of Chinese Medical Sciences, Beijing, 100070 China; 5 College of Life Science, Northeast Forestry University, Harbin, 150040 China; 6 School of Chinese Materia Medica, Tianjin University of Traditional Chinese Medicine, Tianjin, 300193 China

## Abstract

Polyherbal formulations (PHFs) serve as a cornerstone of traditional medical systems worldwide and remain integral to diverse therapeutic practices. The COVID-19 pandemic has reinvigorated scientific interest in PHFs, driving extensive investigations into their composition and pharmacological potential. In this study, we systematically analyzed PHFs across major traditional medical systems, including Traditional Chinese Medicine (TCM), Kampo, Ayurveda, and Unani. To facilitate comprehensive analysis, we developed the Global Polyherbal Formulation Database (GPFD), a repository documenting formulation names, sources, historical evolution, ingredient composition, dosages, medicinal origins, and therapeutic applications. Leveraging this extensive dataset, we conducted a cross-system comparative analysis of PHFs using statistical analysis and data integration approaches. Our investigation focused on plant usage frequency and co-occurrence patterns to uncover underlying principles of formulation design. This study establishes a research framework for PHFs, explores the relationship between species selection, and ecological distributions, and identifies key taxonomic differences in plant utilization across systems. Additionally, we reveal distinct preferences in herbal combinations within each system, highlighting both shared and unique formulation patterns. These findings provide critical insights into the cross-cultural utilization of PHFs and offer a data-driven foundation for integrating traditional botanical knowledge into modern pharmacological research and drug development.

**Supplementary Information:**

The online version contains supplementary material available at 10.1186/s13020-025-01145-7.

## Introduction

Natural products and polyherbal formulations (PHFs) continue to play a significant role in both traditional and modern medical practices [1]. Terms such as"polyherbal formulation,""herbal recipe,""herbal preparations,"and"classical prescription"are commonly used to describe these plant-based therapeutic combinations [2–4]. Following the COVID-19 pandemic, interest in herbal medicines has risen sharply, particularly in developing regions such as Asia, the Middle East, and Africa, where they are valued for their affordability, perceived efficacy, and favorable safety profile [5, 6]. During this process, PHFs have been widely employed and reported to show therapeutic benefits in various regions. Nonetheless, significant controversy persists concerning their scientific validation, safety profiles, and integration within modern regulatory systems [7].

Across countries and regions worldwide, PHFs have been developed based on diverse foundational theories of traditional medical systems and varying geographical contexts, leading to the selection of different medicinal plants and formulation strategies. The origins of PHFs can date back to the earliest recorded medical practices [1]. Ancient physicians systematically explored the therapeutic properties of natural substances through empirical observation and experimentation [8]. As medical knowledge advanced, practitioners in civilizations such as China, Egypt, Greece, and India began formulating compound remedies by combining botanical, mineral, and animal-derived ingredients [1]. Over time, these formulations evolved into standardized preparations tailored to specific ailments within well-established medical frameworks. Refined through centuries of practice and empirical validation, they laid the foundation for enduring traditional medical systems [3]. In China, PHFs have played a pivotal role in the evolution of medical history and remain a cornerstone of modern Chinese medicine [9]. Even with the rise of iatrochemistry, these formulations have retained their significance, physicians continue to integrate traditional medical knowledge with modern practices to develop effective treatment strategies [10, 11]. Successive Chinese dynasties documented standardized PHFs in numerous ancient books, many of which are still widely used in clinical practice today [9]. In recent years, the Chinese government has actively promoted the advancement of these ancient remedies by officially designating select formulations as"Classical Prescription,"streamlining their registration processes [12]. This initiative has revitalized research interest, facilitating deeper exploration of their therapeutic potential.

However, despite the widespread clinical use of PHFs and the growing global interest in their application—particularly in response to public health crises such as the COVID-19 pandemic—the underlying formulation principles, herb selection logic, and therapeutic structures of PHFs across different traditional medical systems remain insufficiently understood. There is an urgent need to establish an integrative platform for cross-system analysis of PHFs. Such a framework would allow for the systematic identification of shared and divergent formulation strategies, reveal recurrent herb combinations and underlying structural features, and provide valuable insights to inform regulatory policies and the integration of traditional medicines into modern healthcare systems. Additionally, it may facilitate the discovery of promising medicinal plants with high therapeutic potential that have yet to be adequately explored.

To support research and development in this field, several publicly accessible databases focusing on Traditional Chinese Medicine (TCM) formulations have been established. Notable examples include The Encyclopedia of Traditional Chinese Medicine (ETCM), Traditional Chinese Medicine Integrative Database (TCMID), Integrated Traditional Chinese Medicine (ITCM), and LTM-TCM, among others. ETCM catalogs 48,442 TCM formulations, 9872 Chinese patent medicines, 2079 medicinal materials, 38,298 chemical constituents, 1040 confirmed or potential drug targets, and 8045 associated diseases, providing a valuable resource for identifying bioactive compounds, facilitating drug discovery, and supporting mechanistic studies of TCM [13]. TCMID 2.0 integrates data on formulations, medicinal herbs, compounds, drugs, diseases, and related targets, enabling network-based analyses of herb-disease and ingredient-target interactions [14]. ITCM offers pharmaco-transcriptomic datasets for 496 active TCM ingredients, supporting research in network pharmacology and novel drug discovery [15]. LTM-TCM consolidates formulations, plant species, chemical constituents, therapeutic targets, and over 1.17 million interactions among TCM components, integrating data from clinical records, ancient texts, and multiple databases [16]. Other noteworthy resources, such as SymMap, and TCMBank, incorporate techniques such as symptom mapping or text-mining other than information integration [17, 18].

Despite these advancements, no existing database systematically specializes in the comparative study of Chinese Classical Prescriptions and PHFs on a global scale. Researchers frequently encounter challenges related to variations in ingredient proportions, unit conversions, dosage inconsistencies, and the accurate identification of medicinal origins when studying traditional PHFs [19]. Most current databases predominantly focus on network pharmacology and mapping symptoms to drug targets and genes [20], while providing limited information on the formulations themselves. This gap highlights the need for a standardized framework to facilitate a comprehensive study of PHFs.

To address this gap, we developed the Global Polyherbal Formulation Database (GPFD), a dedicated platform designed for the standardized documentation and analysis of traditional PHFs [21]. The database systematically compiles detailed information, including formulation names, sources, historical evolution, ingredient composition, dosages, medicinal origins, and associated therapeutic indications across different traditional medical systems. Utilizing this resource, we conducted a comparative analysis to characterize the constituent herbs and formulations, offering insights into their unique features and broader applications.

## Material and method

### Data sources and preprocessing

This study focuses on traditional PHFs, which are composed of multiple plant-based ingredients and have a long history of use in diverse medical traditions. We conducted a review of traditional medical systems from various regions, highlighting region-specific herbal therapies. These regions include China, Korea, Japan, India, the Arabic region, Brazil, North America, ancient Greece, West Africa, and Australia [1–4, 22–24]. To ensure the reliability and relevance of the data, we selected medical systems with continuous documentation and systematically organized records on PHFs, and the collected formulations should be still in use nowadays. Based on these criteria, we included TCM classical prescriptions, Ayurveda from India, Kampo from Japan, and Unani medicine with roots in Arabic traditions.

### The traditional Chinese medicine classical prescription

The TCM classical prescriptions were retrieved from the book *Guidelines for Developing Classical Prescriptions (Jing Dian Ming Fang Kai Fa Zhi Yin)*, which is associated with ISBN 9787030659668. This book recorded 100 classical prescriptions and their development guidelines. It explores the fundamental elements involved in assessing the therapeutic qualities of traditional formulations, detailing the factors relevant to mass production, formulation techniques, and the standards and approaches for quality assessment. Additionally, it addresses clinical positioning issues, determines principles and methods for clinical application, and underscores critical aspects that require attention [25].

### Kampo, Ayurveda, and Unani formulations

For Kampo, we referred to the *Approval Standards for the Manufacturing and Marketing of OTC Kampo Formulations* (*一般用漢方製剤製造販売承認基準について*), a formal collection of formulations supervised by the Pharmaceutical Affairs Bureau of the Japanese Ministry of Health, Labour and Welfare [26]. As the standards were published in Japanese, we selected the edition translated by Xuping Gu which was published by the Shanghai University of Traditional Chinese Medicine in December 1989 [27]. The standards were first published by the Japanese authorities in 1975 and have undergone several revisions, serving as an official guideline for Kampo formulation manufacturing in Japan. The 1989 edition stands out for its professional translations and detailed explanations of formulations included. As no subsequent editions with comparable depth and clarity have been identified, this version was selected as the primary source for our database. For Ayurveda, we retrieved formulations from the *Ayurvedic Pharmacopoeia of India, Part II (Formulations)*, published by the Pharmacopoeia Commission for Indian Medicine & Homoeopathy, Ministry of AYUSH, Government of India. The volumes 1, 2, and 3, published in 2007, 2008, and 2010, were all included [28]. For Unani, we referred to the *Unani Pharmacopoeia of India volumes 1 to 4, Part II (Formulations)*, published by the Pharmacopoeia Commission for Indian Medicine & Homoeopathy, Ministry of AYUSH, Government of India. These volumes were published in 2009, 2010, 2014, and 2019, respectively [29].

### Data processing

To facilitate a comparative evaluation of TCM, Kampo, Ayurveda, and Unani, we adopted a standardized approach to data collection, focusing on formulation names, ingredient composition, source plants, and the respective quantities of each component. Certain non-botanical ingredients, such as honey in Ayurveda, were excluded from subsequent analyses.

For TCM, data were obtained with authorization from the author of *Guidelines for Developing Classical Prescriptions*. Information on Kampo formulations was sourced from publicly available official regulations and a Chinese-Language PDF version of the reference book. The text was processed using optical character recognition (OCR) technology, followed by meticulous manual verification and refinement. Data on Ayurveda and Unani formulations were extracted from publicly accessible PDF documents, with content manually curated and systematically organized.

### Identification of species and species names

Given that all biological materials used in this comparative study are plant-derived, it was essential to accurately identify and standardize the species and their scientific names. However, discrepancies in plant naming conventions across different sources posed a significant challenge—multiple names may refer to the same species, or conversely, the same name might denote different species depending on the context or tradition. To ensure consistency and enable cross-system comparisons, we adopted a unified taxonomic framework. All species names were standardized using the NCBI Taxonomy database [30], with taxonomic classifications cross-referenced through either the Species 2000 China Node or the Catalogue of Life [31, 32]. At the same time, we preserved the original Latin binomials recorded in each source text and made them traceable in the database. This allows users to connect each entry with its historical or system-specific documentation, ensuring both analytical rigor and fidelity to traditional records.

### Prediction of ecologically suitable distributions

The Global Medicinal Plant Geographic Information System (GMPGIS) was employed to analyze the potential habitat of herbs in the database, as well as to compare the ecological status of the plants included in the data. We integrated environmental factors, spatial data, sampling sites, and specific botanical attributes through the GMPGIS platform. By processing these datasets and aligning them with geospatial map layers, each herb included in the study was manually curated. The resulting data were ultimately transformed into visualized maps for comprehensive analysis. The available geographic data for certain species might be inadequate, potentially compromising subsequent analyses. Employing the predicted ecologically suitable distributions could help us to some degree in sidestepping this concern [33]. Additional tools, such as Microsoft Excel and Draw.io, were utilized to ensure systematic data organization and effective visualization.

### Database implementation

The dataset supporting the conclusions of this article is available in the GPFD via https://www.gtmpd.com. The database is publicly accessible for academic use and contains curated data on global polyherbal formulations, categorized by traditional medical system, region, species and formulation. The GPFD was developed using the Django framework version 4.0 [34], with user interfaces implemented using DaisyUI version 4.12 [35]. All data were imported into a MySQL database version 6.0 [36], and systematically structured by data type to support efficient retrieval and analysis. The server infrastructure utilizes uWSGI [37] and Nginx [38] for application deployment and performance optimization.

## Results

### The global polyherbal formulation database

**Fig. 1:**
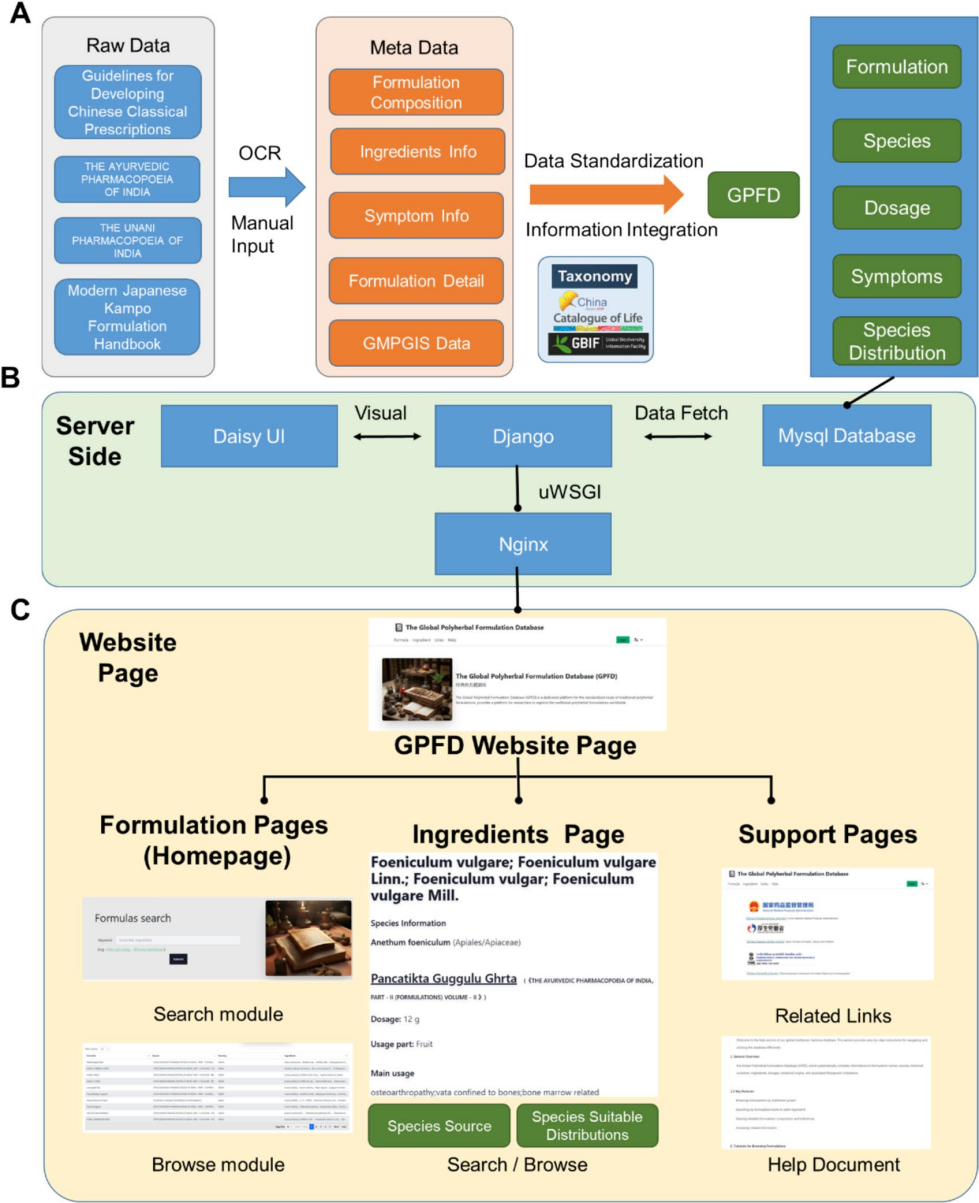
Schematic diagram of the GPFD database. **A** The data process pipeline;** B** The server-side technology of the GPFD site. **C** The pages of the GPFD site include formulations pages, ingredients pages, and support pages. Search and browse modules are also accessible for formulations or ingredients

The GPFD catalogs 612 traditional PHFs from TCM, Kampo, Ayurveda, and Unani medical systems. Each formulation entry in GPFD includes metadata encompassing formulation composition, ingredient details, associated symptoms, formulation descriptions, and GMPGIS-predicted ecologically suitable distributions for nearly all source plants (Fig. 1). This database enables researchers to search and compare formulations based on key attributes such as formulation names, sources, traditional medical systems, and specific ingredients.

**Fig. 2:**
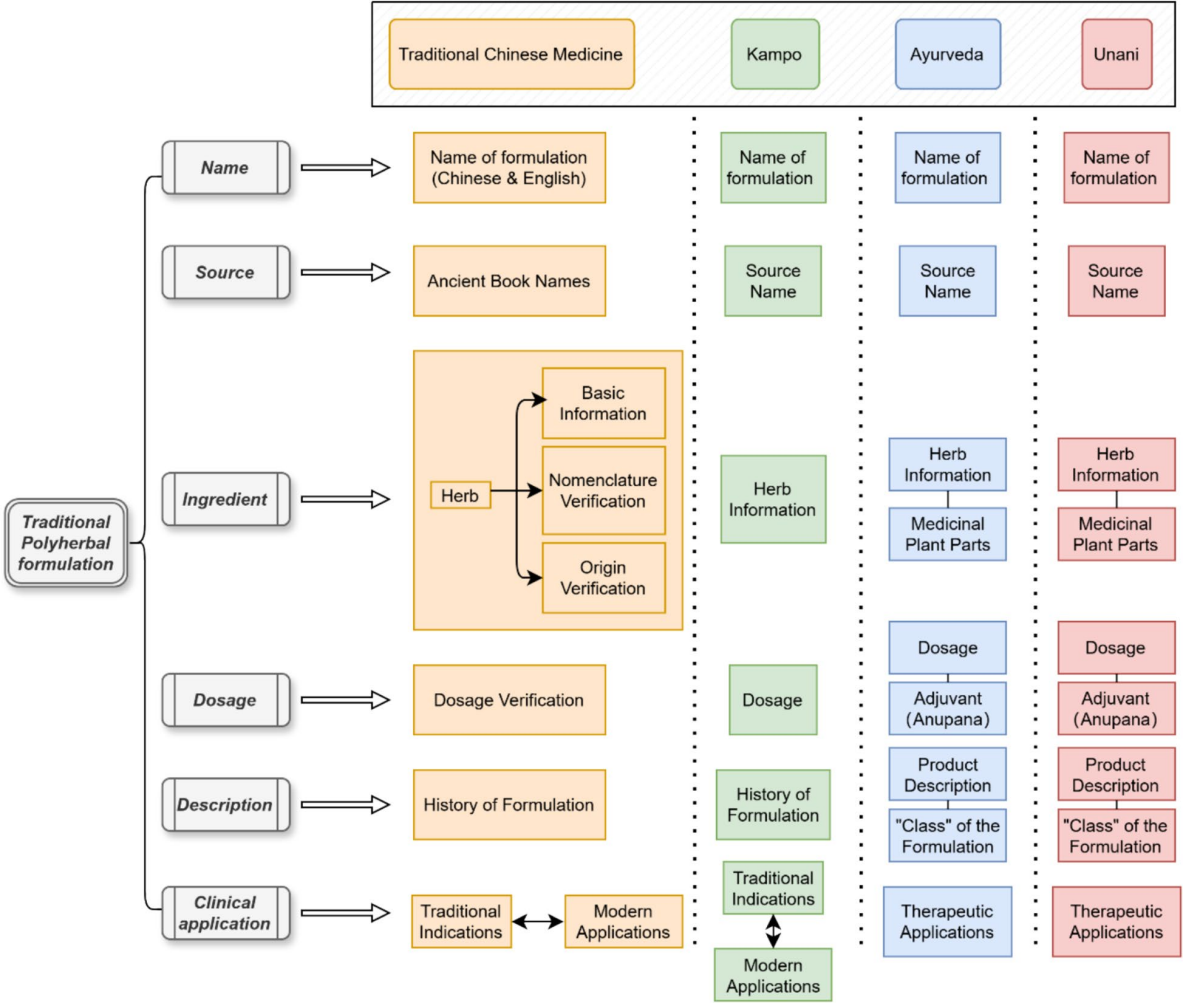
Research framework of traditional polyherbal formulations

To address discrepancies in formulation standards across different traditional medical systems, we established a standardized research framework for PHFs. Each system exhibits distinct characteristics; for instance, TCM emphasizes the origins, processing methods, and historical evolution of medicinal ingredients, whereas Ayurveda provides detailed classifications of its unique therapeutic categories and finished product characteristics [2, 19]. Based on an analysis of 100 TCM classical prescriptions, we developed a unified structural format for each formulation, comprising six key components: name, source, ingredients, dosage, description, and clinical application (Fig. 2).

Using this framework, we conducted a comparative analysis of formulations and plant-derived ingredients, identifying key similarities and differences among the four medical systems. Non-botanical ingredients, including animal-derived substances, minerals, and single compounds, were excluded from the analysis. The dataset includes 100 TCM formulations encompassing 135 herbs, 200 Kampo formulations with 130 herbs, 142 Ayurvedic formulations featuring 239 herbs, and 170 Unani formulations incorporating 272 herbs (Additional File 1: Figure S1). An additional excel file shows the integrated list of PHFs with more detail [see Additional file 2].

### Characteristics of herbs used in traditional medicinal systems

**Fig. 3:**
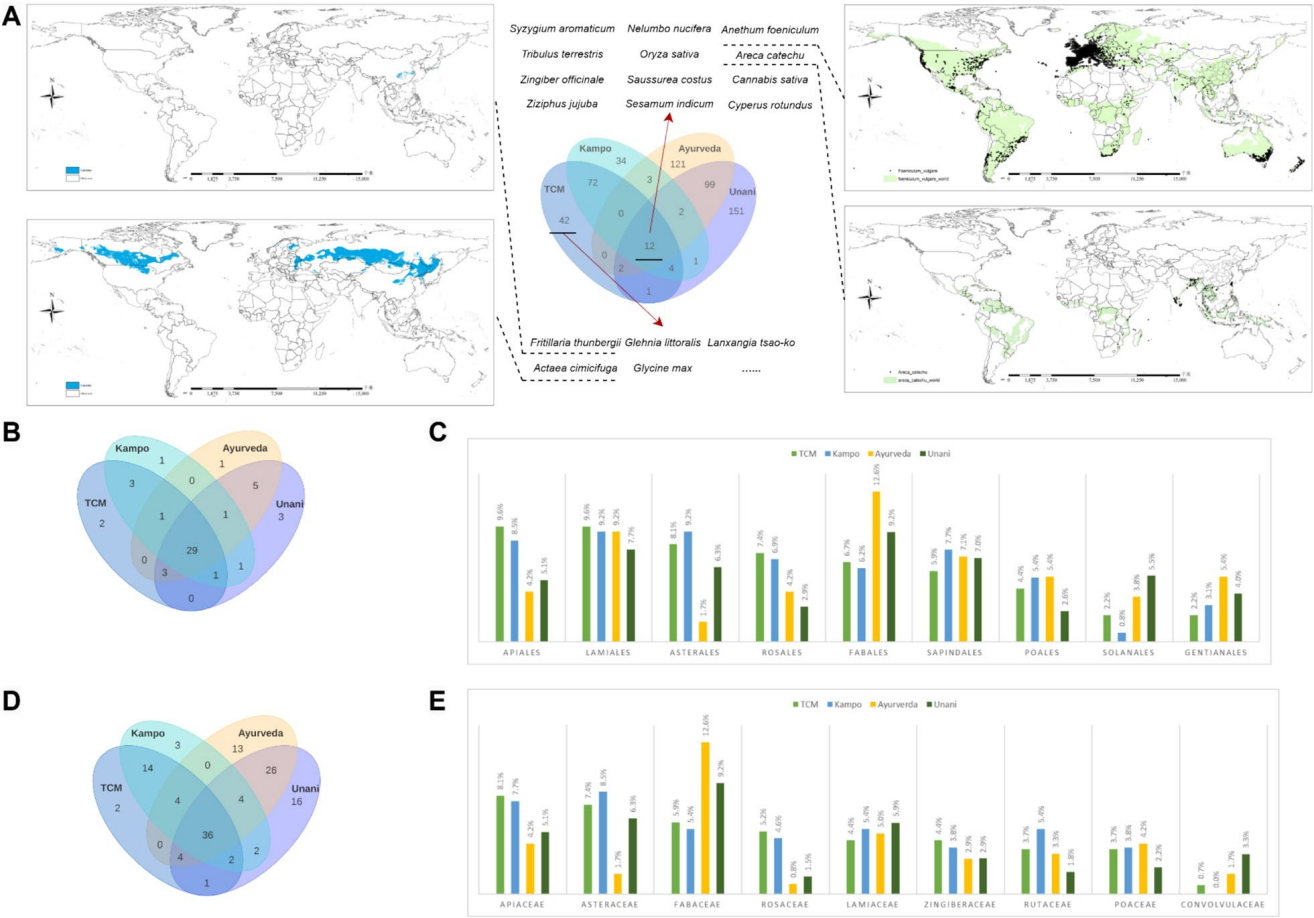
Distribution and overlaps of herbs in TCM, Kampo, Ayurveda, and Unani.** A** Venn plot of overlaps of the herb in TCM, Kampo, Ayurveda, and Unani. Twelve herbs shared by all systems and 5 (out of 42) herbs restricted to TCM were listed, along with the illustration of predicted ecologically suitable distributions of *Fritillaria thunbergia, Actaea cimicifuga, Anethum Foeniculum,* and *Areca catechu*. **B** Overlaps of herb at the order level. **C** Comparison of top five most common orders of herb between TCM, Kampo, Ayurveda and Unani. **D** overlaps of herbs at the family level; **E** comparison of the top five most common families of herbs between TCM, Kampo, Ayurveda, and Unani

A total of 546 herbs were identified, comprising 541 species from the Plantae kingdom and 5 from the Algae kingdom. Only 12 plant species were found to be common across all four medical systems: *Anethum Foeniculum*, *Areca catechu*, *Cannabis sativa*, *Cyperus rotundus*, *Nelumbo nucifera*, *Oryza sativa*, *Saussurea costus*, *Sesamum indicum*, *Syzygium aromaticum*, *Tribulus terrestris*, *Zingiber officinale,* and *Ziziphus jujuba*. In comparison to TCM, 69.2% of the herbs used in Kampo overlap with those in TCM. However, the overlap between TCM and Ayurveda or Unani is significantly lower, with only 5.9% of Ayurvedic herbs and 7.0% of Unani herbs shared with TCM. In contrast, Ayurveda and Unani exhibit a higher degree of similarity, with 42.3% of Unani herbs also present in Ayurveda (Fig. 3A).

To further investigate the ecological distribution of these herbs, we utilized GMPGIS to predict their suitable habitats. All results have been integrated into the database terminal, allowing researchers to access and compare potential ecological niches. Geographic and ecological factors may partially explain why certain herbs are widely used across multiple medical systems, while others are more restricted. For example, *Fritillaria thunbergii* and *Actaea cimicifuga* are exclusively found in TCM. Despite *Actaea cimicifuga* having a broader predicted ecological distribution than *Fritillaria thunbergii*, its habitat is confined to high-latitude regions, which may account for its absence in South Asian and Arabic medical systems. Conversely, *Anethum foeniculum*, which is used in all four systems, thrives in diverse climates and can be cultivated worldwide. However, ecological adaptability is not the sole determinant of an herb’s inclusion in a medical system. For instance, *Areca catechu* is favored to adapt low-latitude regions, and in China, it is found exclusively in a relatively narrow southern area. Despite its limited ecological distribution, it remains a recognized component of Kampo, even though its cultivation in Japan is unlikely (Fig. 3A).

#### Distribution of herbs at different taxonomic levels

We next analyzed the distribution of herbs in four medical systems across taxonomic levels. After conducting a preliminary analysis of the herbs in the phylum, class, order, family, genus, and species, we decided to focus on the levels of order and family as it can balance both broadness and specificity, encompassing the major characteristics of species while also illustrating valid relationships [39].

At the order level, the most frequently represented orders are Apiales and Lamiales in TCM, Lamiales and Asterales in Kampo, and Fabales in both Ayurveda and Unani. Across all four medical systems, 56.9% of the orders are shared. In pairwise comparisons, Kampo and TCM exhibit the highest similarity (91.9%), followed by Ayurveda and TCM (82.5%) and Unani and TCM (88.4%). Notably, Lamiales is well-represented across all four systems, constituting over 7% of the recorded herbs: 9.6% in TCM, 9.2% in Kampo, 9.2% in Ayurveda, and 7.7% in Unani. Other predominant orders include Apiales, Fabales, and Sapindales, each comprising more than 5% of the recorded formulations within at least one system (Fig. 3B, C).

At the family level, the most common families are Apiaceae for TCM, Asteraceae for Kampo, and Fabaceae for both Ayurveda and Unani. Among the four traditional medical systems, 28.3% of the families used in formulations are shared. In pairwise comparisons, Kampo and TCM exhibit a similarity of 89.2%, Ayurveda and TCM show a similarity of 49.4%, and Unani and TCM share a similarity of 47.3%. Fabaceae, Lamiaceae, and Apiaceae are relatively frequent families, each accounting for more than 4% of the formulations. The proportions of these families within each system are as follows: TCM (5.9%, 4.4%, 8.1%), Kampo (5.4%, 5.4%, 7.7%), Ayurveda (12.6%, 5.0%, 4.2%), and Unani (9.2%, 5.9%, 5.1%) (Fig. 3D, E).

#### Frequencies of herb usage at the plant family level

**Fig. 4:**
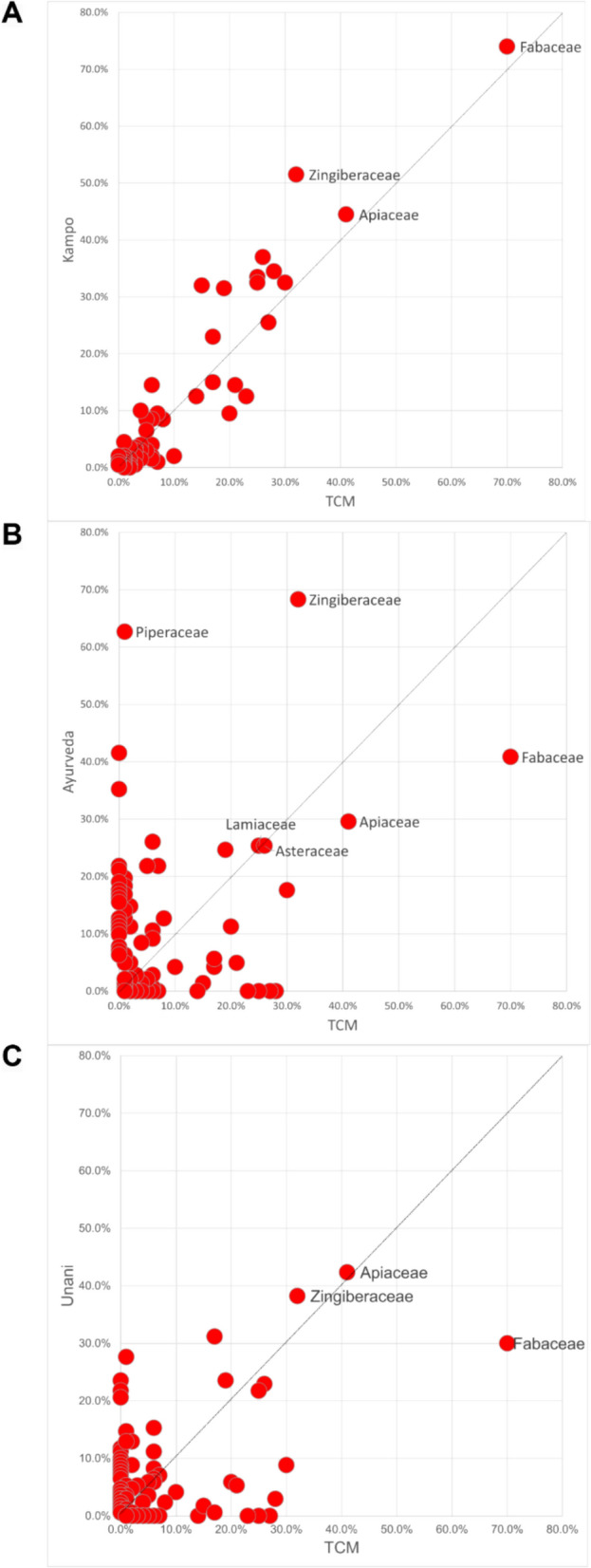
Frequencies of herb usage at the family level. **A** Comparison of herb frequencies in formulations between TCM and Kampo. **B** Comparison of herb frequencies in formulations between TCM and Ayurveda. **C** Comparison of herb frequencies in formulations between TCM and Unani

Recognizing the diversity of herb usage in traditional medicine systems, we used TCM as the baseline to analyze the frequency of plant species at the taxonomic levels of family and order. We then compared these frequencies with those found in Kampo, Ayurveda, and Unani (Fig. 4; Additional file 1: Figure S2). Since the family of plants provides a more detailed reflection of regional characteristics and resource utilization when comparing plants from different regions, we primarily present the results at the family level [39].

TCM and Kampo exhibit similar distributions in the frequency of plant family usage. Fabaceae appears in 70% of TCM formulations and 74% of Kampo formulations, encompassing 15 plant species, of which four are shared between the two systems. Apiaceae is present in 41% of TCM formulations and 44.5% of Kampo formulations, while Zingiberaceae occurs in 32% of TCM formulations and 51.5% of Kampo formulations (Fig. 4A).

In comparison, TCM and Ayurveda demonstrate a more dispersed selection of medicinal plant families, though certain families exhibit similar frequencies in both systems. At the family level, Apiaceae appears in 41% of TCM formulations and 29.6% of Ayurveda formulations, spanning 22 different herbs; however, *Anethum Foeniculum* is the only shared species. Conversely, Piperaceae is present in 62.7% of Ayurvedic formulations but only in 1.0% of TCM formulations, highlighting a divergence in plant selection between these systems (Fig. 4B).

When comparing TCM with Unani, apart from a few plant families showing similar usage frequencies, the two systems exhibit minimal overlap in botanical selection. Apiaceae is included in 41% of TCM formulations and 42.4% of Unani formulations, comprising 24 plant species, with *Anethum foeniculum* as the only shared herb. Zingiberaceae appears in 32% of TCM formulations and 38.2% of Unani formulations, encompassing 12 plant species, of which only *Curcuma longa* and *Zingiber officinale* are common in both systems (Fig. 4C).

### Comparison of herb combinations among traditional medicinal systems

On average, each TCM formulation consists of 6.8 herbs, each Kampo formulation contains 7.3 herbs, each Ayurveda formulation includes 12.9 herbs, and each Unani formulation has 8.5 herbs (Additional file 1: Figure S1). While certain herbs in TCM, Kampo, and Ayurveda formulations exhibit higher usage frequencies, the distribution of herbs in Unani medicine is more dispersed (Additional file 1: Fig. S3).

In TCM and Kampo, the most frequently used herb is *Glycyrrhiza uralensis*, appearing in 60% and 71.5% of formulations, respectively. In Ayurveda, *Piper longum* is the most prevalent herb, found in 54.2% of formulations, while *Zingiber officinale* is the most frequently used herb in Unani, occurring in 27.6% of formulations (Table 1).

**Table 1:** Proportion of most common herbs and herb pairs

Top five herbs used in the formulation			Top 5 herb pairs within the formulation		
	Herb	Proportion	Herb 1	Herb 2	Proportion
TCM	*Glycyrrhiza uralensis*	60.0%	*Glycyrrhiza uralensis*	*Panax ginseng*	20.0%
	*Paeonia lactiflora*	27.0%	*Glycyrrhiza uralensis*	*Zingiber officinale*	19.0%
	*Angelica sinensis*	26.0%	*Glycyrrhiza uralensis*	*Paeonia lactiflora*	18.0%
	*Panax ginseng*	26.0%	*Angelica sinensis*	*Glycyrrhiza uralensis*	16.0%
	*Zingiber officinale*	26.0%	*Glycyrrhiza uralensis*	*Wolfiporia cocos*	16.0%
Kampo	*Glycyrrhiza uralensis*	71.5%	*Glycyrrhiza uralensis*	*Zingiber officinale*	41.00%
	*Zingiber officinale*	49.0%	*Glycyrrhiza uralensis*	*Ziziphus jujuba*	29.00%
	*Wolfiporia cocos*	33.5%	*Zingiber officinale*	*Ziziphus jujuba*	28.50%
	*Paeonia lactiflora*	32.0%	*Cinnamomum aromaticum*	*Glycyrrhiza uralensis*	25.50%
	*Ziziphus jujuba*	32.0%	*Glycyrrhiza uralensis*	*Paeonia lactiflora*	24.00%
Ayurveda	*Piper longum*	54.2%	*Piper longum*	*Zingiber officinale*	38.7%
	*Zingiber officinale*	47.2%	*Piper longum*	*Piper nigrum*	31.7%
	*Terminalia chebula*	38.0%	*Emblica officinalis*	*Terminalia chebula*	31.0%
	*Piper nigrum*	36.6%	*Piper nigrum*	*Zingiber officinale*	30.3%
	*Emblica officinalis*	35.2%	*Terminalia bellirica*	*Terminalia chebula*	27.5%
Unani	*Zingiber officinale*	27.6%	*Emblica officinalis*	*Terminalia chebula*	14.7%
	*Terminalia chebula*	22.9%	*Terminalia bellirica*	*Terminalia chebula*	13.5%
	*Emblica officinalis*	20.6%	*Emblica officinalis*	*Terminalia bellirica*	13.5%
	*Piper nigrum*	19.4%	*Piper nigrum*	*Zingiber officinale*	12.4%
	*Rosa x damascena*	18.8%	*Piper longum*	*Zingiber officinale*	10.0%

The widespread use of *Glycyrrhiza uralensis* in TCM and Kampo can be attributed to its non-toxic and edible nature, as well as its common role in"herb pairs"within TCM [40]. This trend persists in Kampo medicine, where most formulations originate from ancient Chinese medical texts, as documented in the *Modern Japanese Kampo Formulation Handbook* [27]. Similarly, *Piper longum* holds significant importance in Ayurveda, functioning as both a key therapeutic agent and a bioenhancer that enhances the efficacy and bioavailability of other medicinal compounds [41]. Given its diverse pharmacological properties, *Piper longum* is widely employed in managing various clinical conditions. In the context of Unani medicine, the frequent use of *Zingiber officinale* and *Terminalia chebula* is well-grounded. *Zingiber officinale*, the most commonly used herb in this system, enjoys widespread application across the globe. Recognized for its safety profile, *Zingiber officinale* is associated with minimal and non-significant side effects while exhibiting strong antioxidant activity [42]. *Terminalia chebula*, on the other hand, has held a highly esteemed position in South Asia since ancient times. Contemporary pharmacological studies have identified its involvement in at least 23 distinct biological activities, including antioxidant and anti-lipid peroxidation effects, underscoring its broad therapeutic potential [43].

These findings indicate that PHFs consist of diverse combinations of herbs, with an uneven distribution of their usage. To explore this further, we conducted an additional analysis on herb combinations within formulations.

#### Overall patterns of herb combinations in traditional medicinal systems

In TCM, the five most common herb pairings within formulations are *Glycyrrhiza uralensis* with *Panax ginseng* (20%), *Glycyrrhiza uralensis* with *Zingiber officinale* (19%), *Glycyrrhiza uralensis* with *Paeonia lactiflora* (18%), *Angelica sinensis* with *Glycyrrhiza uralensis* (16%), and *Glycyrrhiza uralensis* with *Poria cocos* (16%). Given that *Glycyrrhiza uralensis* appears in 60% of TCM formulations, its frequent pairing with other herbs is unsurprising.

In Kampo, the most common herb combination is *Glycyrrhiza uralensis* and *Zingiber officinale*, occurring in 41% of formulations. In Ayurveda, the five most frequent pairings are *Piper longum* with *Zingiber officinale* (39%), *Piper longum* with *Piper nigrum* (32%), *Emblica officinalis* with *Terminalia chebula* (31%), *Piper nigrum* with *Zingiber officinale* (30%), and *Terminalia bellirica* with *Terminalia chebula* (28%). In Unani, the most common pairing is *Emblica officinalis* and *Terminalia chebula*, appearing in 14.7% of formulations (Table 1).

**Fig. 5:**
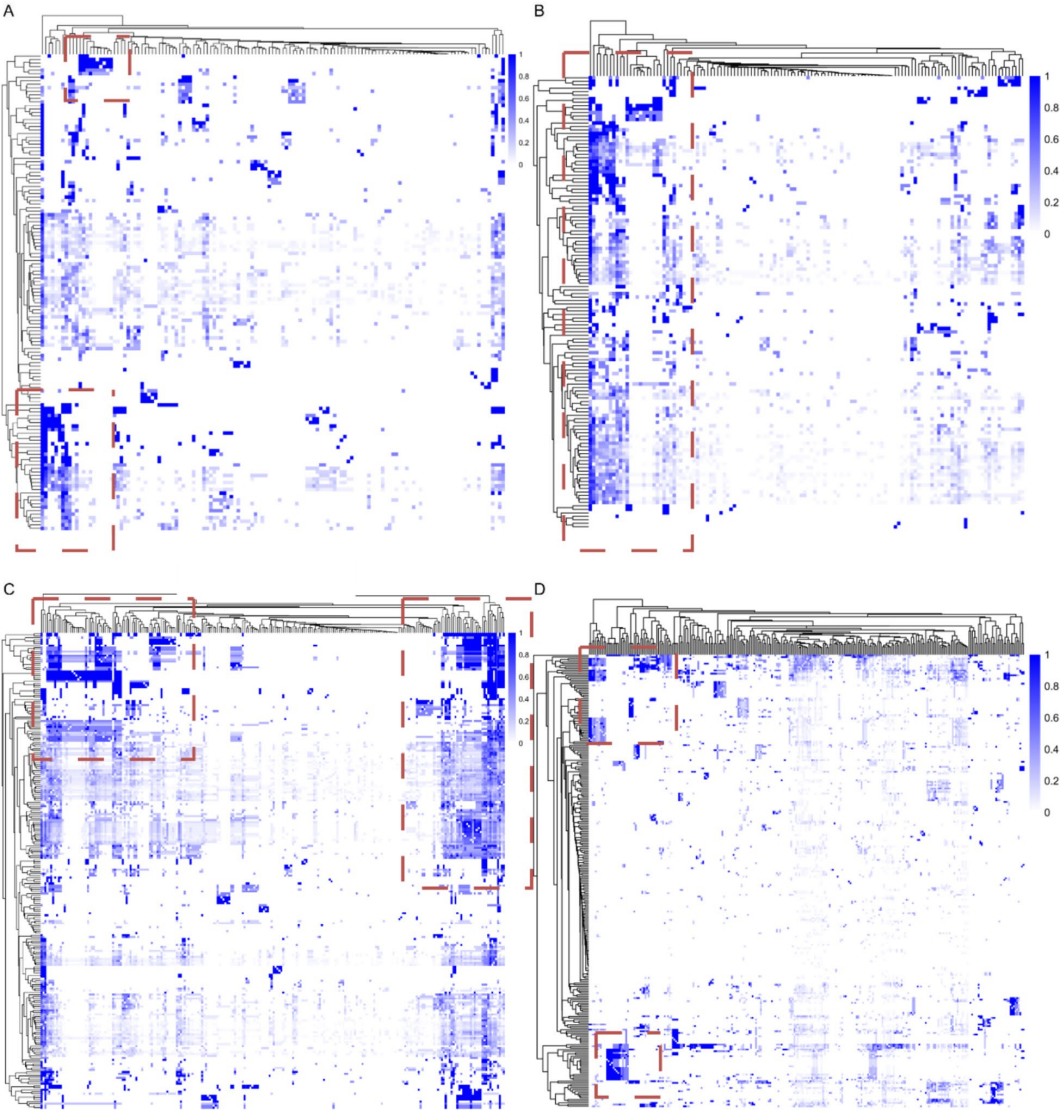
Heatmap of co-occurrence scores for every herb combination in **A** TCM; **B** Kampo;** C** Ayurveda; **D** Unani. In cases where herb *a* is always co-exist with herb *b*, the co-occurrence score of *a* on *b* is defined as 1. The *dashed-line rectangles* indicate regions where herb pairs exhibit a high co-occurrence score

To further analyze herb co-occurrence, we generated a heatmap comparing pairwise combinations, assessing the dependency of one herb’s presence on another (Fig. 5). In each heatmap, the x-axis and y-axis represent individual herbs within a medical system, while their intersection reflects the probability of co-occurrence, graded by color intensity. For example, in TCM, *Glycyrrhiza uralensis* and *Panax ginseng* co-occur with a probability of 33.3% when *Glycyrrhiza uralensis* is present and 76.9% when *Panax ginseng* appears (Additional file 1: Table S1).

The distribution of TCM pairwise combinations in the heatmap exhibits a block-like clustering pattern, where specific herb pairs frequently co-occur in formulations. In contrast, Kampo displays a strip-like distribution, indicating that while certain herbs are used frequently, they do not form distinct, fixed pairings as seen in TCM. Ayurveda follows a pattern similar to TCM but with larger and more pronounced block-like clusters, suggesting that a substantial number of herb pairings appear recurrently in formulations. In Unani, the distribution is more evenly spread, with most pairwise combinations lacking strong co-occurrence patterns. However, some small clusters indicate that certain herbs still tend to be paired together (Fig. 5).

#### Instances of herb combinations

Some specific herb combinations exhibit a high probability of co-occurrence. In our dataset, when analyzing combinations with a frequency exceeding 5%, we identified the following patterns:

In TCM, *Saposhnikovia divaricata* and *Glycyrrhiza uralensis* co-occur in 10% of formulations, when a formulation contains *Saposhnikovia divaricata*, *Glycyrrhiza uralensis* always presents. Similar dependencies were observed in five other combinations, including *Angelica pubescens* always need *Glycyrrhiza uralensis*, *Angelica dahurica* pairs *Ligusticum chuanxiong* and *Glycyrrhiza uralensis, Gentiana macrophylla* occur with *Glycyrrhiza uralensis*, and *Fritillaria thunbergii* with *Gardenia jasminoides*. In Kampo, two strong co-occurrence patterns emerged: *Mentha canadensis* always appears with *Glycyrrhiza uralensis*, and *Saposhnikovia divaricata* consistently follows *Glycyrrhiza uralensis* in formulations. Ayurveda exhibits even more pronounced dependency patterns, with 12 combinations showing high co-occurrence. Among them, two pairs demonstrate strong dependency from both sides, meaning the presence of one plant guarantees the presence of each other: *Uraria picta* with *Pleurolobus gangeticus* and *Oroxylum indicum* with *Stereospermum suaveolens*. In contrast, no such absolute co-occurrence patterns were observed in Unani formulations. When we broadened the analysis to include combinations where one herb had an occurrence probability of over 80% alongside another (within combinations exceeding 5% frequency), the results showed variation across systems: TCM had 18 such combinations, Kampo had 16, Ayurveda exhibited a markedly higher number with 65, while Unani contained only 3 (Additional file 1: Tables S2–S5).

#### Unexpected ecologically suitable distribution of common combinations

Overall, 37.4% of identical two-herb combinations are shared between TCM and Kampo, whereas only 12.7% overlap with Ayurveda and Unani. Beyond these, the overlap among other systems is minimal (Additional file 1: Tables S2–S5).

Among the pairwise combinations across different medical systems, three combinations appear consistently in all four traditions: *Saussurea costus* and *Zingiber officinale*, *Cyperus rotundus* and *Saussurea costus*, and *Syzygium aromaticum* with *Zingiber officinale*.

A straightforward explanation for the persistent occurrence of these combinations—and the four herbs involved—could be their shared geographic distribution. However, ecological suitability predictions based on GMPGIS suggest otherwise. *Cyperus rotundus* has the broadest predicted ecological range, spanning temperate, subtropical, and tropical regions across all continents. Yet, its combination with *Zingiber officinale*—another herb with a similarly wide predicted range—is not among the universally shared pairs. Meanwhile, *Syzygium aromaticum* is restricted to tropical regions, and *Saussurea costus* has an even more limited range, mainly within the Himalayas.

**Fig. 6:**
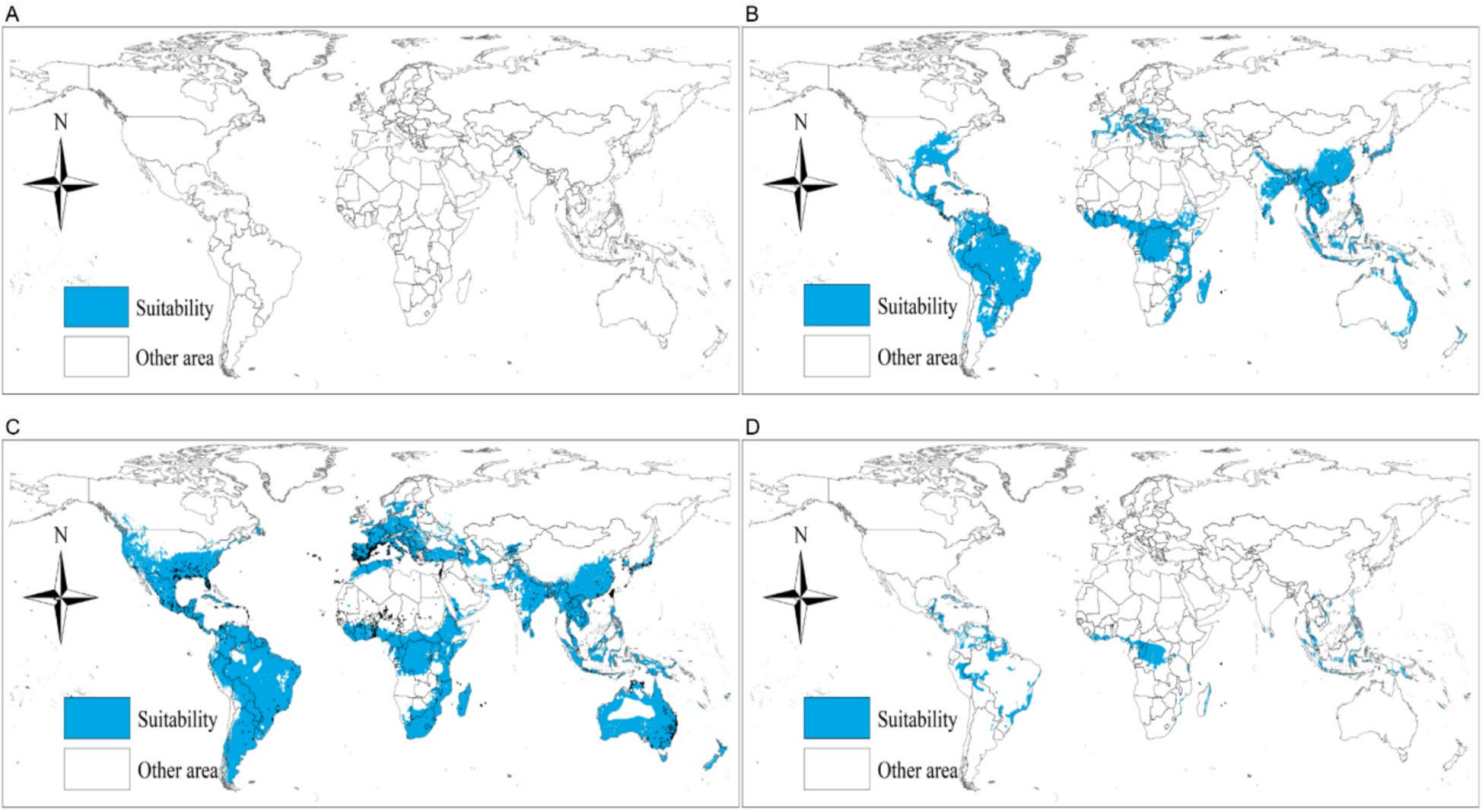
Predicted ecologically suitable distributions of **A** *Saussurea costus*; **B** *Zingiber officinale*; **C** *Cyperus rotundus*; **D** *Syzygium aromaticum*

This discrepancy between ecological distribution and medicinal use suggests that geography alone does not dictate herb selection in traditional medical systems. Other factors, such as historical transmission, cultural preferences, or pharmacological properties, may influence the selection of combinations in traditional medical practice. Based on the records, China, India, Arabic countries, and Japan were connected through the Silk Road and Maritime Silk Road*. Cyperus rotundus*, *Saussurea costus*, and *Syzygium aromaticum* were introduced into China in large quantities around the sixth century CE via those trade routes. *Syzygium aromaticum* is a representative example of an imported medicine, originally sourced from Southeast Asia and coastal regions of Africa. Although it is now cultivated domestically in China, its initial introduction occurred primarily through the Maritime Silk Road. *Saussurea costus*, produced in both the Arab and Indian regions, was historically a major trade commodity and was eventually cultivated in southwestern China. *Cyperus rotundus* is also frequently mentioned in historical records concerning commercial exchanges [44]. In traditional Chinese classification, such herbs were categorized as “Fragrant Medicines,” a term referring to substances with aromatic qualities and therapeutic effects, commonly used in both incense and medical formulations and imported from foreign countries (Fig. 6) [45].

## Discussion

Our findings reveal similarities between TCM and Kampo, largely due to their shared historical and cultural foundations in medicinal plant use. However, each system has evolved distinct characteristics shaped by regional and cultural adaptations [3]. While the specific plant species used across traditional medical systems vary significantly, this disparity diminishes at lower taxonomic levels, such as order and family. This convergence suggests underlying common principles in the selection and application of botanical resources, providing valuable insights into the pharmacological and therapeutic strategies that these systems share. This study analyzed plant combinations within formulations, revealing intriguing patterns across different medical systems. While certain plants are frequently used due to their unique status within their respective traditions, some herbal combinations exhibit strong associations within formulations. Such analyses could provide novel insights into the compositional principles underlying traditional medical systems and contribute to the development of innovative therapeutic approaches in modern medicine.

The current database encompasses four major traditional systems—TCM, Kampo, Ayurveda, and Unani—selected for their significance and relative accessibility of data. However, the dataset is predominantly derived from East and South Asia, with Ayurveda and Unani data heavily sourced from Indian governmental repositories. Historically, Unani medicine originated from ancient Greek traditions, later transmitted through Persia during the medieval period, and subsequently influenced Europe and South Asia [46]. Yet, the present data may not fully capture the diversity or current practices of Unani medicine in regions such as the Middle East or North Africa. Limitations such as language barriers and the lack of publicly accessible digital resources from these areas contribute to this regional bias. Similarly, the primary sources of traditional PHFs in TCM, Kampo, and Ayurveda are derived largely from authoritative classical texts and standardized compendia. While these sources ensure consistency and traceability, they may not comprehensively reflect the full breadth and regional variability of traditional medical practice, particularly in folk or orally transmitted formulations.

In this study, we utilized both the existing and potential ecological environments of various species when applying GMPGIS for analysis and comparison, rather than relying solely on the available herb sampling sites. GMPGIS was developed to analyze environmental information within ecologically suitable regions, thereby guiding the conservation and introduction of medicinal plants, incorporating a range of ecological factors [33]. Relying exclusively on sampling site data could lead to an underestimation of species’ ecological distribution. We acknowledge the possibility of overestimation, particularly regarding the ecological distribution of certain species, which may deviate from actual conditions. Overall, this approach provides a valuable method to address species sampling limitations and offers practical guidance for future species production and cultivation.

We acknowledge this study is limited by the absence of symptom-specific formulation analyses. Although our database includes descriptions of the symptoms in each formulation, these descriptions lack standardized terminology and are deeply rooted in the unique diagnostic frameworks of their respective medical systems. Addressing this limitation will require the establishment of a unified standard for symptom descriptions and a systematic approach to mapping the relationships between symptoms and formulations, which represents a critical direction for future research.

This study not only provides a foundational framework for the comparative analysis of PHFs across major traditional medical systems but also opens several avenues for application and future development. The establishment of the GPFD offers substantial potential for both pharmaceutical innovation and global health equity. In the context of drug discovery, the GPFD can serve as a hypothesis-generating platform. Systematizing traditional formulation knowledge enables the identification of understudied plant taxa and recurrent herb combinations that exhibit therapeutic promise yet remain insufficiently explored in modern pharmacological research. These findings can be cross-referenced with emerging front-end screening technologies, such as high-throughput screening (HTS) and artificial intelligence (AI)-driven target prediction. Recent advancements in G protein-coupled receptor (GPCR)-based drug screening strategies exemplify how traditional medicinal knowledge, when digitized and structured, can accelerate early-stage lead identification by narrowing the chemical search space to bioactive phytocompounds with long-standing empirical use [47, 48]. In this context, GPFD offers a robust backend of traditional knowledge that can be paired with modern screening technologies to accelerate early-stage drug discovery.

On another hand, the GPFD also presents implications for countries with limited health care expenses. Documenting and comparing affordable, locally available herbal solutions, can inform the development of integrative health interventions that are both cost-effective and culturally relevant. In settings where access to conventional pharmaceuticals is limited, such a resource may support the rational adoption of safe, evidence-informed traditional therapies as primary or adjunctive treatment strategies. Moreover, these novel therapeutic strategies and PHFs can be further optimized through geo-environmental suitability insights provided by GMPGIS. By leveraging such data, it becomes feasible to prioritize the cultivation and development of medicinal plants that are ecologically adapted to local conditions, thereby promoting region-specific, sustainable healthcare solutions.

The utility of the GPFD hinges on its continued expansion, technological integration, and contextual adaptation. Future efforts should prioritize the inclusion of PHFs from underrepresented traditional systems and enrich the database with ecological, ethnopharmacological, and pharmacokinetic metadata. These additions will strengthen the cultural and scientific validity of cross-system comparisons. Advanced technologies such as natural language processing (NLP) and machine learning will be essential for scaling data acquisition and enabling more nuanced analyses of herb pairings, therapeutic structures, and formulation principles. Moreover, these tools can facilitate the exploration of more complex combinations involving three or more herbs, thus uncovering higher-order synergistic patterns that are often overlooked in pairwise analyses.

## Conclusion

PHFs are integral to the evolution and practice of traditional medical systems worldwide, offering a unique blend of historical wisdom and therapeutic potential. The establishment of GPFD provides a platform for researchers to explore traditional PHFs worldwide. By constructing a standardized database and conducting a comparative analysis of TCM, Kampo, Ayurveda, and Unani, this study provides a foundational framework for understanding the shared and unique characteristics of these systems. Our findings suggest that, despite regional and cultural differences, these medical traditions converge on core principles of botanical resource utilization. This alignment highlights the potential for cross-cultural integration and knowledge exchange to enhance modern pharmacological research and therapeutic applications. Looking ahead, further efforts to standardize symptom descriptions and expand the scope of the dataset will facilitate deeper insights into global traditional PHFs. Additionally, exploring multi-herb combinations will uncover the complexities of traditional formulations, advancing their integration into modern medical practices and drug discovery. By bridging traditional wisdom with modern science, this work contributes to the broader goal of fostering a more holistic and sustainable approach to global healthcare.

## Supplementary Information

Additional file 1.

Additional file 2.

## Data Availability

The datasets during and/or analysed during the current study available from the corresponding author on reasonable request.

## Abbreviations

PHFs: Polyherbal Formulations
TCM: Traditional Chinese Medicine
GPFD: the Global Polyherbal Formulation Database
ETCM: The Encyclopedia of Traditional Chinese Medicine
TCMID: Traditional Chinese Medicine Integrative Database
ITCM: Integrated Traditional Chinese Medicine
OCR: Optical Character Recognition
GMPGIS: The Global Medicinal Plant Geographic Information System
HTS: High-Throughput Screening
AI: Artificial Intelligence
GPCR: G protein-coupled receptor
NLP: Natural Language Processing

## Abbreviations

PHFs: Polyherbal Formulations
TCM: Traditional Chinese Medicine
GPFD: the Global Polyherbal Formulation Database
ETCM: The Encyclopedia of Traditional Chinese Medicine
TCMID: Traditional Chinese Medicine Integrative Database
ITCM: Integrated Traditional Chinese Medicine
OCR: Optical Character Recognition
GMPGIS: The Global Medicinal Plant Geographic Information System
HTS: High-Throughput Screening
AI: Artificial Intelligence
GPCR: G protein-coupled receptor
NLP: Natural Language Processing
