# Supplementary material for: Comparative analysis of Chinese classical prescriptions and global traditional polyherbal formulations: insights from the database of global polyherbal formulation (GPFD)
Source: Chin Med. 2025 Oct 7;20:169. doi: 10.1186/s13020-025-01145-7 (PMC12502377; doi:10.1186/s13020-025-01145-7)
Supplement: Supplementary file 1 — Additional file 1. [file 13020_2025_1145_MOESM1_ESM.docx]

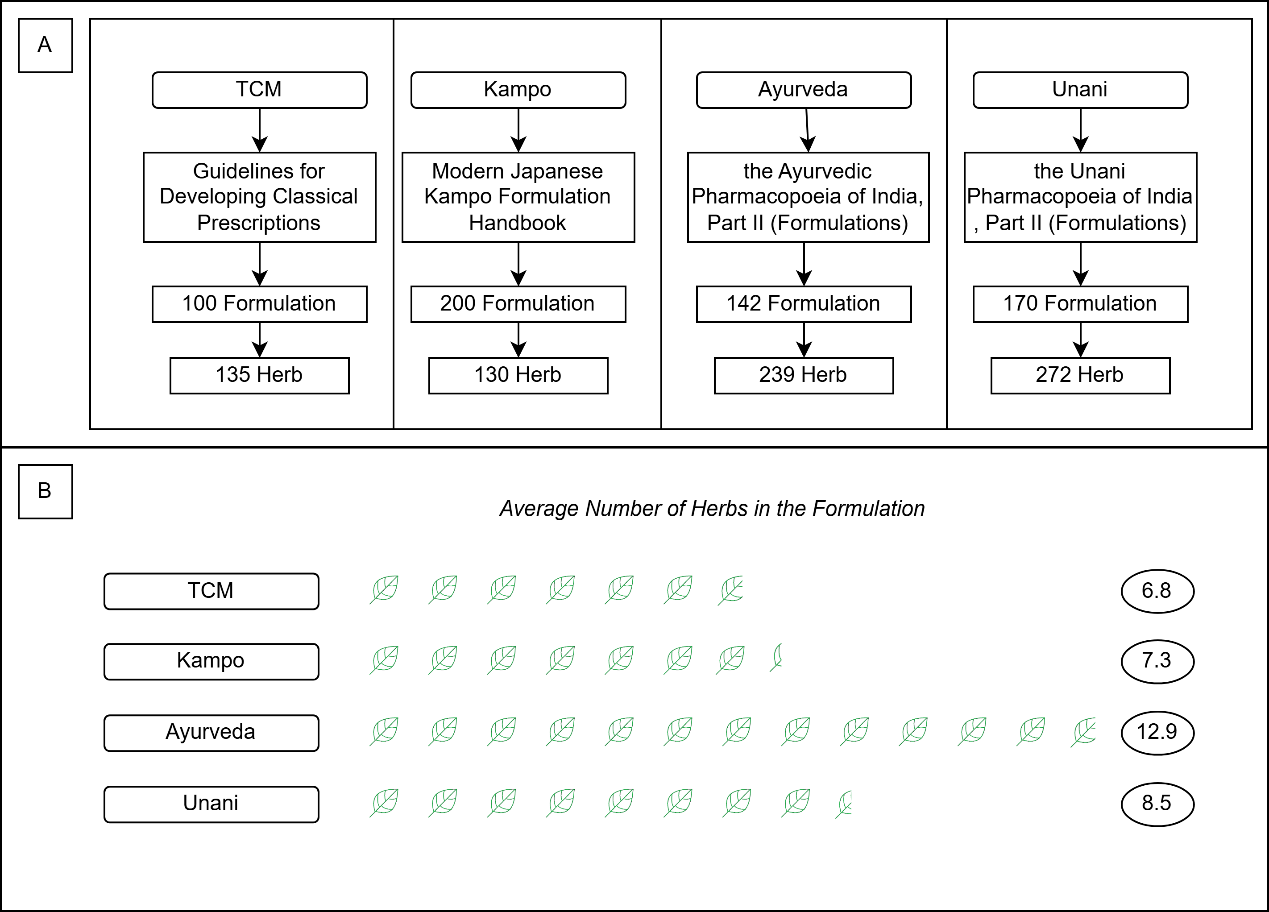


**Figure S1 A** the overview of data selected in comparison study **B** Average number of herbs contained in TCM, Kampo, Ayurveda and Unani formulation

**
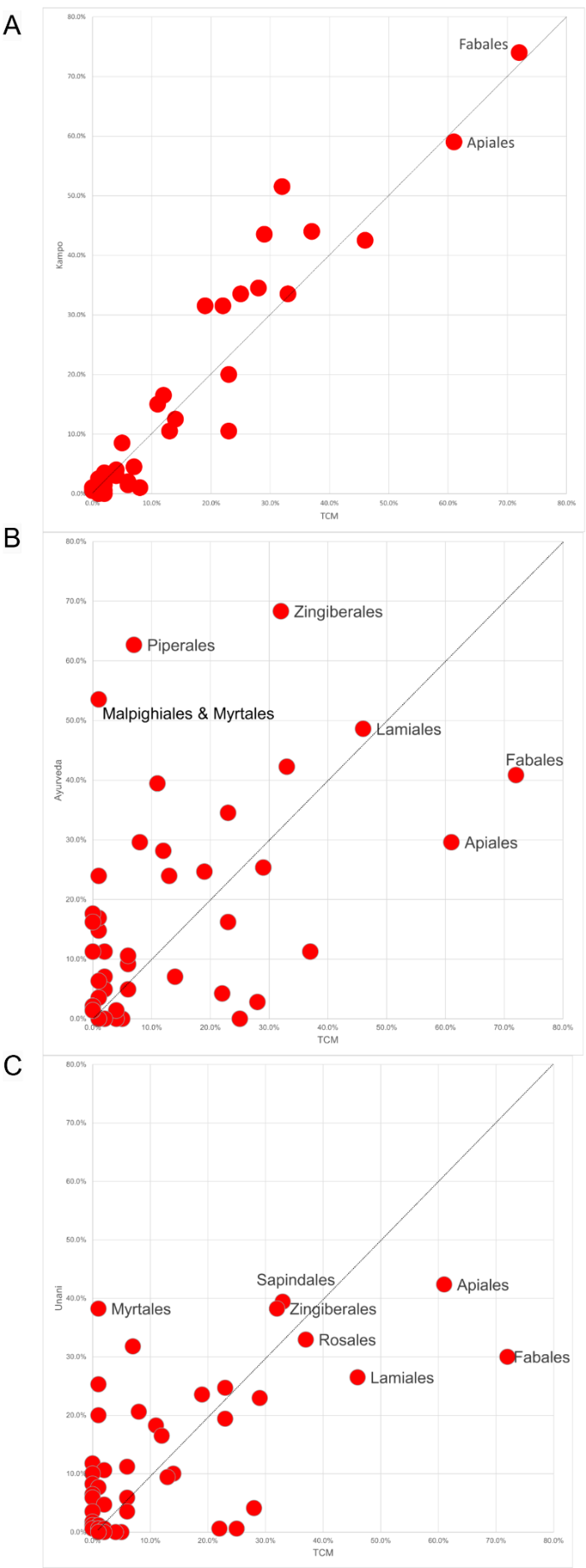
**

**Figure S2** Proportions of herb usage at the Order level. **A** Herb proportion in formulations when comparing TCM with Kampo; **B** Herb proportion in formulations when comparing TCM with Ayurveda; **C** Herb proportion in formulations when comparing TCM with Unani.


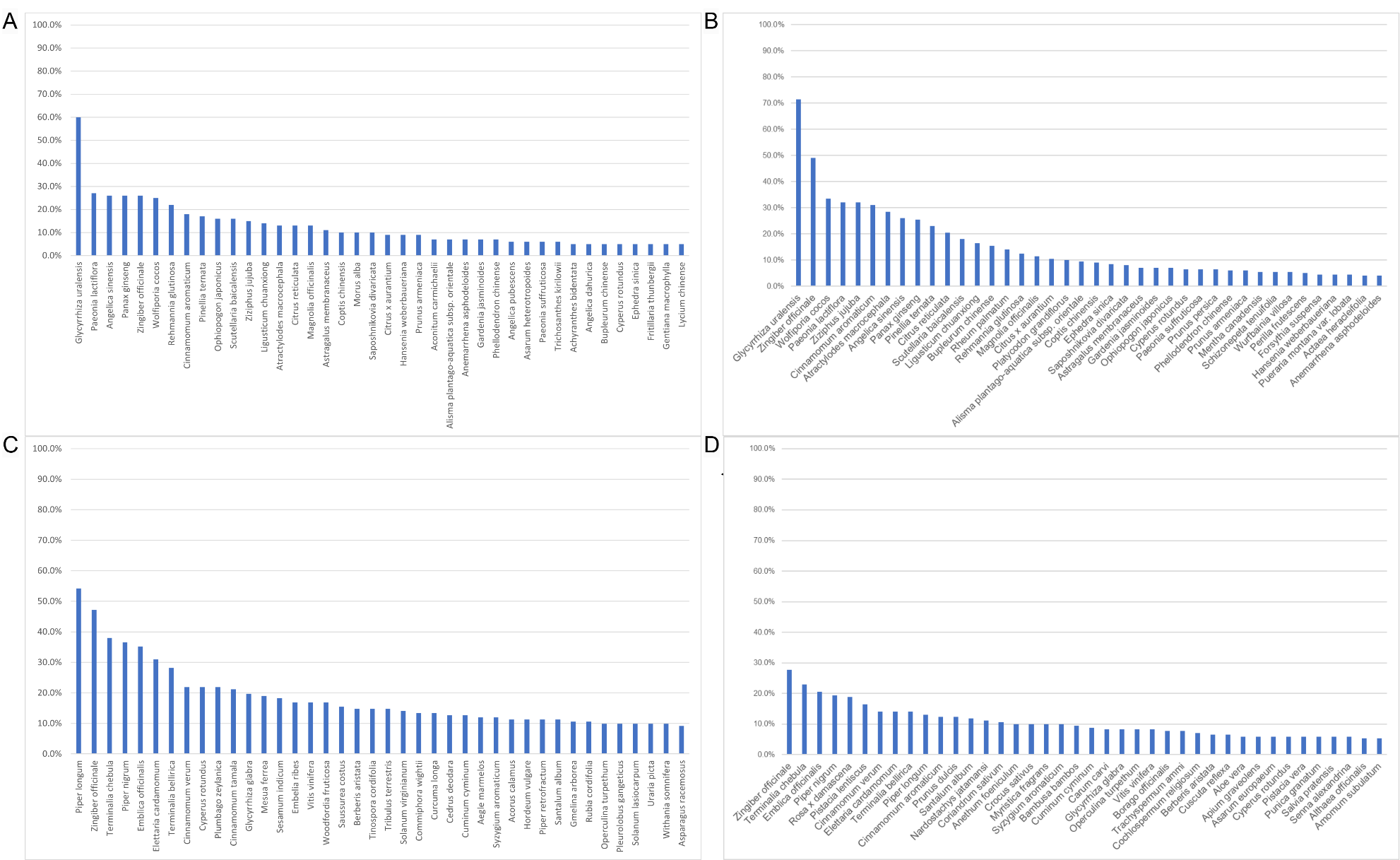


**Figure S3** Frequency of herb occurred in formulation: the top 40 **A** TCM **B** Kampo **C** Ayurveda **D** Unani

| Pairwise combination: co-occurrence probability | | | |  | |  |  |  |
| --- | --- | --- | --- | --- | --- | --- | --- | --- |
|  | Herb 1 | Herb 2 | Pairwise Frequency | | Dependency of herb1 on herb 2 | | Dependency of herb2 on herb 1 |  |
| TCM | *Saposhnikovia divaricata* | *Glycyrrhiza uralensis* | 10.0% | | 100.0% | | 16.7% |  |
|  | *Citrus reticulata* | *Glycyrrhiza uralensis* | 12.0% | | 92.3% | | 20.0% |  |
|  | *Hansenia weberbaueriana* | *Glycyrrhiza uralensis* | 8.0% | | 88.9% | | 13.3% |  |
|  | *Scutellaria baicalensis* | *Glycyrrhiza uralensis* | 14.0% | | 87.5% | | 23.3% |  |
|  | *Ligusticum chuanxiong* | *Glycyrrhiza uralensis* | 12.0% | | 85.7% | | 20.0% |  |
|  | *Angelica pubescens* | *Ligusticum chuanxiong* | 5.0% | | 83.3% | | 35.7% |  |
|  | *Angelica pubescens* | *Hansenia weberbaueriana* | 5.0% | | 83.3% | | 55.6% |  |
|  | *Asarum heterotropoides* | *Glycyrrhiza uralensis* | 5.0% | | 83.3% | | 8.3% |  |
|  | *Angelica pubescens* | *Saposhnikovia divaricata* | 5.0% | | 83.3% | | 50.0% |  |
|  | *Paeonia suffruticosa* | *Paeonia lactiflora* | 5.0% | | 83.3% | | 18.5% |  |
| Kampo | *Saposhnikovia divaricata* | *Glycyrrhiza uralensis* | 8.0% | | 100.0% | | 11.0% |  |
|  | *Mentha canadensis* | *Glycyrrhiza uralensis* | 5.5% | | 100.0% | | 7.6% |  |
|  | *Cyperus rotundus* | *Glycyrrhiza uralensis* | 6.0% | | 92.3% | | 8.3% |  |
|  | *Pinellia ternata* | *Zingiber officinale* | 21.0% | | 91.3% | | 39.3% |  |
|  | *Bupleurum chinense* | *Glycyrrhiza uralensis* | 14.0% | | 90.3% | | 19.3% |  |
|  | *Ephedra sinica* | *Glycyrrhiza uralensis* | 8.5% | | 89.5% | | 11.7% |  |
|  | *Asarum heterotropoides* | *Glycyrrhiza uralensis* | 3.5% | | 87.5% | | 4.8% |  |
|  | *Anemarrhena asphodeloides* | *Glycyrrhiza uralensis* | 3.5% | | 87.5% | | 4.8% |  |
|  | *Actaea heracleifolia* | *Glycyrrhiza uralensis* | 3.5% | | 87.5% | | 4.8% |  |
|  | *Astragalus membranaceus* | *Glycyrrhiza uralensis* | 6.0% | | 85.7% | | 8.3% |  |
| Ayurveda | *Pleurolobus gangeticus* | *Uraria picta* | 9.9% | | 100.0% | | 100.0% |  |
|  | *Stereospermum suaveolens* | *Oroxylum indicum* | 9.2% | | 100.0% | | 100.0% |  |
|  | | *Oroxylum indicum* | *Aegle marmelos* | 9.2% | | 100.0% | | 76.5% |
|  | | *Stereospermum suaveolens* | *Aegle marmelos* | 9.2% | | 100.0% | | 76.5% |
|  | | *Cinnamomum verum* | *Elettaria cardamomum* | 21.8% | | 100.0% | | 70.5% |
|  | | *Cinnamomum tamala* | *Elettaria cardamomum* | 21.1% | | 100.0% | | 68.2% |
|  | | *Clerodendrum phlomidis* | *Oroxylum indicum* | 5.6% | | 100.0% | | 61.5% |
|  | | *Clerodendrum phlomidis* | *Stereospermum suaveolens* | 5.6% | | 100.0% | | 61.5% |
|  | | *Myristica fragrans* | *Syzygium aromaticum* | 7.0% | | 100.0% | | 58.8% |
|  | | *Piper cubeba* | *Syzygium aromaticum* | 6.3% | | 100.0% | | 52.9% |
| Unani | | *Terminalia bellirica* | *Terminalia chebula* | 13.5% | | 95.8% | | 59% |
|  | | *Terminalia bellirica* | *Emblica officinalis* | 13.5% | | 95.8% | | 65.7% |
|  | *Carum carvi* | *Zingiber officinale* | 7.6% | | 92.9% | | 27.7% |  |

**Table S1** Pairwise combination: top 10 co-occurrence probability within TCM, Kampo, Ayurveda and Unani (with pairwise frequency over 5% and dependence rate over 80%)

| Pairwise combination: co-occurrence probability | |  |  |  |
| --- | --- | --- | --- | --- |
| Herb 1 | Herb 2 | Pairwise Frequency | Dependency of herb1 on herb 2 | Dependency of herb2 on herb 1 |
| *Glycyrrhiza uralensis* | *Panax ginseng* | 20.0% | 33.3% | 76.9% |
| *Glycyrrhiza uralensis* | *Zingiber officinale* | 19.0% | 31.7% | 73.1% |
| *Glycyrrhiza uralensis* | *Paeonia lactiflora* | 18.0% | 30.0% | 66.7% |
| *Angelica sinensis* | *Glycyrrhiza uralensis* | 16.0% | 61.5% | 26.7% |
| *Glycyrrhiza uralensis* | *Wolfiporia cocos* | 16.0% | 26.7% | 64.0% |
| *Cinnamomum aromaticum* | *Glycyrrhiza uralensis* | 14.0% | 77.8% | 23.3% |
| *Glycyrrhiza uralensis* | *Scutellaria baicalensis* | 14.0% | 23.3% | 87.5% |
| *Angelica sinensis* | *Rehmannia glutinosa* | 12.0% | 46.2% | 54.5% |
| *Glycyrrhiza uralensis* | *Ligusticum chuanxiong* | 12.0% | 20.0% | 85.7% |
| *Glycyrrhiza uralensis* | *Ziziphus jujuba* | 12.0% | 20.0% | 80.0% |
| *Angelica sinensis* | *Paeonia lactiflora* | 12.0% | 46.2% | 44.4% |
| *Citrus reticulata* | *Glycyrrhiza uralensis* | 12.0% | 92.3% | 20.0% |
| *Glycyrrhiza uralensis* | *Pinellia ternata* | 12.0% | 20.0% | 70.6% |
| *Zingiber officinale* | *Ziziphus jujuba* | 12.0% | 46.2% | 80.0% |
| *Glycyrrhiza uralensis* | *Ophiopogon japonicus* | 11.0% | 18.3% | 68.8% |
| *Paeonia lactiflora* | *Rehmannia glutinosa* | 11.0% | 40.7% | 50.0% |
| *Panax ginseng* | *Zingiber officinale* | 11.0% | 42.3% | 42.3% |
| *Glycyrrhiza uralensis* | *Saposhnikovia divaricata* | 10.0% | 16.7% | 100.0% |
| *Atractylodes macrocephala* | *Wolfiporia cocos* | 10.0% | 76.9% | 40.0% |
| *Angelica sinensis* | *Ligusticum chuanxiong* | 10.0% | 38.5% | 71.4% |
| *Atractylodes macrocephala* | *Glycyrrhiza uralensis* | 10.0% | 76.9% | 16.7% |
| *Pinellia ternata* | *Zingiber officinale* | 10.0% | 58.8% | 38.5% |
| *Glycyrrhiza uralensis* | *Rehmannia glutinosa* | 9.0% | 15.0% | 40.9% |
| *Citrus reticulata* | *Wolfiporia cocos* | 9.0% | 69.2% | 36.0% |
| *Ligusticum chuanxiong* | *Paeonia lactiflora* | 9.0% | 64.3% | 33.3% |
| *Glycyrrhiza uralensis* | *Hansenia weberbaueriana* | 8.0% | 13.3% | 88.9% |
| *Glycyrrhiza uralensis* | *Morus alba* | 8.0% | 13.3% | 80.0% |
| *Wolfiporia cocos* | *Zingiber officinale* | 8.0% | 32.0% | 30.8% |
| *Paeonia lactiflora* | *Panax ginseng* | 8.0% | 29.6% | 30.8% |
| *Cinnamomum aromaticum* | *Zingiber officinale* | 8.0% | 44.4% | 30.8% |
| *Atractylodes macrocephala* | *Paeonia lactiflora* | 7.0% | 53.8% | 25.9% |
| *Panax ginseng* | *Pinellia ternata* | 7.0% | 26.9% | 41.2% |
| *Panax ginseng* | *Ziziphus jujuba* | 7.0% | 26.9% | 46.7% |
| *Pinellia ternata* | *Ziziphus jujuba* | 7.0% | 41.2% | 46.7% |
| *Panax ginseng* | *Wolfiporia cocos* | 7.0% | 26.9% | 28.0% |

**Table S2** Pairwise combination: Part of co-occurrence probability within TCM (top 35 most common pairwise)

| Pairwise combination: co-occurrence probability | |  |  |  |
| --- | --- | --- | --- | --- |
| Herb 1 | Herb 2 | Pairwise Frequency | Dependency of herb1 on herb 2 | Dependency of herb2 on herb 1 |
| *Glycyrrhiza uralensis* | *Zingiber officinale* | 41.00% | 56.60% | 76.60% |
| *Glycyrrhiza uralensis* | *Ziziphus jujuba* | 29.00% | 40.00% | 84.10% |
| *Zingiber officinale* | *Ziziphus jujuba* | 28.50% | 53.30% | 82.60% |
| *Cinnamomum aromaticum* | *Glycyrrhiza uralensis* | 25.50% | 79.70% | 35.20% |
| *Glycyrrhiza uralensis* | *Paeonia lactiflora* | 24.00% | 33.10% | 72.70% |
| *Glycyrrhiza uralensis* | *Wolfiporia cocos* | 23.00% | 31.70% | 68.70% |
| *Atractylodes macrocephala* | *Glycyrrhiza uralensis* | 23.00% | 80.70% | 31.70% |
| *Pinellia ternata* | *Zingiber officinale* | 21.00% | 91.30% | 39.30% |
| *Glycyrrhiza uralensis* | *Panax ginseng* | 20.50% | 28.30% | 80.40% |
| *Wolfiporia cocos* | *Zingiber officinale* | 20.50% | 61.20% | 38.30% |
| *Atractylodes macrocephala* | *Wolfiporia cocos* | 20.00% | 70.20% | 59.70% |
| *Panax ginseng* | *Zingiber officinale* | 19.00% | 74.50% | 35.50% |
| *Angelica sinensis* | *Glycyrrhiza uralensis* | 19.00% | 73.10% | 26.20% |
| *Cinnamomum aromaticum* | *Zingiber officinale* | 19.00% | 59.40% | 35.50% |
| *Glycyrrhiza uralensis* | *Pinellia ternata* | 18.50% | 25.50% | 80.40% |
| *Paeonia lactiflora* | *Zingiber officinale* | 18.50% | 56.10% | 34.60% |
| *Cinnamomum aromaticum* | *Paeonia lactiflora* | 18.50% | 57.80% | 56.10% |
| *Atractylodes macrocephala* | *Zingiber officinale* | 17.50% | 61.40% | 32.70% |
| *Citrus reticulata* | *Glycyrrhiza uralensis* | 17.50% | 85.40% | 24.10% |
| *Citrus reticulata* | *Zingiber officinale* | 15.50% | 75.60% | 29.00% |
| *Angelica sinensis* | *Paeonia lactiflora* | 15.50% | 59.60% | 47.00% |
| *Cinnamomum aromaticum* | *Ziziphus jujuba* | 15.00% | 46.90% | 43.50% |
| *Panax ginseng* | *Pinellia ternata* | 14.00% | 54.90% | 60.90% |
| *Glycyrrhiza uralensis* | *Scutellaria baicalensis* | 14.00% | 19.30% | 75.70% |
| *Bupleurum chinense* | *Glycyrrhiza uralensis* | 14.00% | 90.30% | 19.30% |
| *Citrus reticulata* | *Wolfiporia cocos* | 14.00% | 68.30% | 41.80% |
| *Panax ginseng* | *Ziziphus jujuba* | 13.50% | 52.90% | 39.10% |
| *Atractylodes macrocephala* | *Citrus reticulata* | 13.50% | 47.40% | 65.90% |
| *Paeonia lactiflora* | *Ziziphus jujuba* | 13.00% | 39.40% | 37.70% |
| *Angelica sinensis* | *Atractylodes macrocephala* | 13.00% | 50.00% | 45.60% |
| *Pinellia ternata* | *Ziziphus jujuba* | 12.50% | 54.30% | 36.20% |
| *Panax ginseng* | *Wolfiporia cocos* | 12.00% | 47.10% | 35.80% |
| *Glycyrrhiza uralensis* | *Ligusticum chuanxiong* | 12.00% | 16.60% | 72.70% |
| *Atractylodes macrocephala* | *Panax ginseng* | 12.00% | 42.10% | 47.10% |
| *Wolfiporia cocos* | *Ziziphus jujuba* | 11.50% | 34.30% | 33.30% |

**Table S3** Pairwise combination: Part of co-occurrence probability within Kampo (top 35 most common pairwise)

| Pairwise combination: co-occurrence probability | |  |  |  |
| --- | --- | --- | --- | --- |
| Herb 1 | Herb 2 | Pairwise Frequency | Dependency of herb1 on herb 2 | Dependency of herb2 on herb 1 |
| *Piper longum* | *Zingiber officinale* | 38.7% | 71.4% | 82.1% |
| *Piper longum* | *Piper nigrum* | 31.7% | 58.4% | 86.5% |
| *Emblica officinalis* | *Terminalia chebula* | 31.0% | 88.0% | 81.5% |
| *Piper nigrum* | *Zingiber officinale* | 30.3% | 82.7% | 64.2% |
| *Terminalia bellirica* | *Terminalia chebula* | 27.5% | 97.5% | 72.2% |
| *Emblica officinalis* | *Terminalia bellirica* | 26.8% | 76.0% | 95.0% |
| *Piper longum* | *Terminalia chebula* | 24.6% | 45.5% | 64.8% |
| *Emblica officinalis* | *Piper longum* | 23.2% | 66.0% | 42.9% |
| *Terminalia chebula* | *Zingiber officinale* | 22.5% | 59.3% | 47.8% |
| *Cinnamomum verum* | *Elettaria cardamomum* | 21.8% | 100.0% | 70.5% |
| *Elettaria cardamomum* | *Piper longum* | 21.8% | 70.5% | 40.3% |
| *Cinnamomum tamala* | *Elettaria cardamomum* | 21.1% | 100.0% | 68.2% |
| *Cinnamomum tamala* | *Cinnamomum verum* | 19.0% | 90.0% | 87.1% |
| *Emblica officinalis* | *Zingiber officinale* | 19.0% | 54.0% | 40.3% |
| *Piper longum* | *Plumbago zeylanica* | 18.3% | 33.8% | 83.9% |
| *Piper longum* | *Terminalia bellirica* | 17.6% | 32.5% | 62.5% |
| *Cinnamomum verum* | *Piper longum* | 16.9% | 77.4% | 31.2% |
| *Piper nigrum* | *Terminalia chebula* | 16.9% | 46.2% | 44.4% |
| *Elettaria cardamomum* | *Mesua ferrea* | 16.2% | 52.3% | 85.2% |
| *Cinnamomum tamala* | *Piper longum* | 16.2% | 76.7% | 29.9% |
| *Plumbago zeylanica* | *Zingiber officinale* | 16.2% | 74.2% | 34.3% |
| *Cyperus rotundus* | *Piper longum* | 15.5% | 71.0% | 28.6% |
| *Elettaria cardamomum* | *Terminalia chebula* | 15.5% | 50.0% | 40.7% |
| *Terminalia bellirica* | *Zingiber officinale* | 15.5% | 55.0% | 32.8% |
| *Elettaria cardamomum* | *Piper nigrum* | 15.5% | 50.0% | 42.3% |
| *Emblica officinalis* | *Piper nigrum* | 15.5% | 44.0% | 42.3% |
| *Elettaria cardamomum* | *Emblica officinalis* | 14.8% | 47.7% | 42.0% |
| *Elettaria cardamomum* | *Zingiber officinale* | 14.8% | 47.7% | 31.3% |
| *Embelia ribes* | *Piper longum* | 14.8% | 87.5% | 27.3% |
| *Cinnamomum verum* | *Mesua ferrea* | 14.1% | 64.5% | 74.1% |
| *Embelia ribes* | *Terminalia chebula* | 13.4% | 79.2% | 35.2% |
| *Embelia ribes* | *Zingiber officinale* | 13.4% | 79.2% | 28.4% |
| *Mesua ferrea* | *Piper longum* | 13.4% | 70.4% | 24.7% |
| *Cinnamomum verum* | *Piper nigrum* | 13.4% | 61.3% | 36.5% |
| *Piper nigrum* | *Plumbago zeylanica* | 13.4% | 36.5% | 61.3% |

**Table S4** Pairwise combination: Part of co-occurrence probability within Ayurveda (top 35 most common pairwise)

| Pairwise combination: co-occurrence probability | |  |  |  |
| --- | --- | --- | --- | --- |
| Herb 1 | Herb 2 | Pairwise Frequency | Dependency of herb1 on herb 2 | Dependency of herb2 on herb 1 |
| *Emblica officinalis* | *Terminalia chebula* | 14.70% | 71.40% | 64.10% |
| *Terminalia bellirica* | *Terminalia chebula* | 13.50% | 95.80% | 59.00% |
| *Emblica officinalis* | *Terminalia bellirica* | 13.50% | 65.70% | 95.80% |
| *Piper nigrum* | *Zingiber officinale* | 12.40% | 63.60% | 44.70% |
| *Piper longum* | *Zingiber officinale* | 10.00% | 77.30% | 36.20% |
| *Cinnamomum verum* | *Pistacia lentiscus* | 8.20% | 58.30% | 50.00% |
| *Carum carvi* | *Zingiber officinale* | 7.60% | 92.90% | 27.70% |
| *Rosa x damascena* | *Santalum album* | 7.60% | 40.60% | 65.00% |
| *Elettaria cardamomum* | *Syzygium aromaticum* | 7.10% | 50.00% | 70.60% |
| *Cinnamomum verum* | *Nardostachys jatamansi* | 7.10% | 50.00% | 63.20% |
| *Cinnamomum verum* | *Elettaria cardamomum* | 7.10% | 50.00% | 50.00% |
| *Piper longum* | *Piper nigrum* | 7.10% | 54.50% | 36.40% |
| *Elettaria cardamomum* | *Zingiber officinale* | 6.50% | 45.80% | 23.40% |
| *Emblica officinalis* | *Rosa x damascena* | 6.50% | 31.40% | 34.40% |
| *Cinnamomum aromaticum* | *Cinnamomum verum* | 6.50% | 52.40% | 45.80% |
| *Cinnamomum aromaticum* | *Zingiber officinale* | 6.50% | 52.40% | 23.40% |
| *Rosa x damascena* | *Terminalia chebula* | 6.50% | 34.40% | 28.20% |
| *Myristica fragrans* | *Syzygium aromaticum* | 6.50% | 64.70% | 64.70% |
| *Syzygium aromaticum* | *Zingiber officinale* | 6.50% | 64.70% | 23.40% |
| *Cinnamomum aromaticum* | *Piper nigrum* | 6.50% | 52.40% | 33.30% |
| *Cinnamomum aromaticum* | *Elettaria cardamomum* | 5.90% | 47.60% | 41.70% |
| *Nardostachys jatamansi* | *Pistacia lentiscus* | 5.90% | 52.60% | 35.70% |
| *Elettaria cardamomum* | *Pistacia lentiscus* | 5.90% | 41.70% | 35.70% |
| *Cinnamomum aromaticum* | *Pistacia lentiscus* | 5.90% | 47.60% | 35.70% |
| *Cinnamomum verum* | *Piper nigrum* | 5.90% | 41.70% | 30.30% |
| *Myristica fragrans* | *Zingiber officinale* | 5.90% | 58.80% | 21.30% |
| *Cinnamomum verum* | *Zingiber officinale* | 5.90% | 41.70% | 21.30% |
| *Cinnamomum aromaticum* | *Nardostachys jatamansi* | 5.30% | 42.90% | 47.40% |
| *Bambusa bambos* | *Rosa x damascena* | 5.30% | 56.30% | 28.10% |
| *Cinnamomum verum* | *Crocus sativus* | 5.30% | 37.50% | 52.90% |
| *Piper nigrum* | *Syzygium aromaticum* | 5.30% | 27.30% | 52.90% |
| *Coriandrum sativum* | *Rosa x damascena* | 5.30% | 50.00% | 28.10% |
| *Cinnamomum aromaticum* | *Syzygium aromaticum* | 5.30% | 42.90% | 52.90% |
| *Elettaria cardamomum* | *Nardostachys jatamansi* | 5.30% | 37.50% | 47.40% |
| *Cinnamomum verum* | *Syzygium aromaticum* | 5.30% | 37.50% | 52.90% |

**Table S5** Pairwise combination: Part of co-occurrence probability within Unani (top 35 most common pairwise)
